# Preserving the quality of life: nutrition in the ICU

**DOI:** 10.1186/s13054-019-2415-8

**Published:** 2019-06-14

**Authors:** Pierre Singer

**Affiliations:** Critical Care and Institute for Nutrition Research, Rabin Medical Center, Beilinson Hospital, Sackler School of Medicine, Tel Aviv University, Jabotinsky St, 49100 Petah Tikva, Israel

## Abstract

Critically ill patients require adequate nutritional support to meet energy requirements both during and after intensive care unit (ICU) stay to protect against severe catabolism and prevent significant deconditioning. ICU patients often suffer from chronic critical illness causing an increase in energy expenditure, leading to proteolysis and related muscle loss. Careful supplementation and modulation of caloric and protein intake can avoid under- or overfeeding, both associated with poorer outcomes. Indirect calorimetry is the preferred method for assessing resting energy expenditure and the appropriate caloric and protein intake to counter energy and muscle loss. Physical exercise may have favorable effects on muscle preservation and should be considered even early in the hospital course of a critically ill patient. After liberation from the ventilator or during non-invasive ventilation, oral intake should be carefully evaluated and, in case of severe dysphagia, should be avoided and replaced by enteral of parenteral nutrition. Upon transfer from the ICU to the ward, adequate nutrition remains essential for long-term rehabilitation success and continued emphasis on sufficient nutritional supplementation in the ward is necessary to avoid a suboptimal nutritional state.

## Background

The ICU course of patients surviving a critical illness is relatively short, with clinical improvement achieved within 3 to 7 days in the ICU. Notwithstanding the improvement, a subset of patients progress to a chronic critical illness characterized by low-grade persistent inflammation and protein catabolism, a state referred to as Persistent Inflammatory Catabolism Syndrome (PICS) [1]. The quality of life of PICS patients may be substantially impaired, suffering from a significant decrease in functionality (as evidenced by limited 6 min walk distance) as well as impaired reintegration to pre-hospitalization life [2]. Substantial muscle loss, frailty, and sarcopenia are associated with decreased quality of life at 6 and 12 months post-ICU, as well as an increase in organ failure and mortality [3]. The consistent decrease in-hospital mortality achieved over the past years has yielded a threefold increase in ICU survivors with PICS requiring rehabilitation for decreased functional status [4]. Adequate nutritional support in the ICU setting can alter the post-hospital course of these patients, and recent guidelines have been published outlining recommendations for ICU nutrition in this patient population [5]. This review will address the current guideline-based methods for providing adequate nutritional substrates in both early and late phases of ICU hospitalization, as well as the importance of early mobilization and exercise and post-extubation nutritional support.

### Catabolic response to stress

After injury or infection, Cuthbertson described a brief “ebb” phase associated with a decrease in cardiac output and body temperature, followed by an extended “flow” phase associated with an increase in energy expenditure and marked catabolism [6]. The flow phase consists of an early period (approximately 48 h) and a late period (the subsequent 5 to 7 days) [5]. If the patient fails to fully recover in the late period, the flow phase is followed by a chronic critical illness phase which may continue for weeks (PICS), which is associated with increased resting energy expenditure (REE) and severe catabolism.

The nutrients (principally glucose) required for combating wound/injury/infection, in addition to that required by the brain, kidneys, and hematopoeitic system, are derived in part from proteolysis and lipolysis. Conversion of amino acids and glycerol to glucose generally occurs by gluconeogenesis and glycolysis. During the chronic critical illness phase, however, insulin resistance associated with hyperglycemia is common, and nutrient production relies primarily upon proteolysis and lipolysis. A retrospective cohort study conducted at our institution demonstrated that after 2 days of lower energy expenditure (approximately 1650 kcal/d), REE reaches a plateau around 2000 kcal/day [7].

Protein breakdown may reach 12 to 16 g of nitrogen/day within days of admission and can increase up to 30 g of nitrogen/day in certain cases [8]. This loss of nitrogen is associated with a substantial loss of muscle mass. The endogenous production of nutrition substrates through proteolysis and lipolysis to supply sufficient glucose to the body are minimally affected by exogenous supply of substrates through diet. Therefore, during this phase of endogenous substrate production, ICU staff should be cautious not to administer full nutritional therapy that might lead to overfeeding.

### Energy requirements and prescription

Overnutrition and, more commonly, undernutrition are very frequent in the ICU, and the time to reach target energy requirements may be prolonged [9]. The NutritionDay ICU audit found that it takes 1 week to reach 1500 kcal intake in most ICUs in the world [9]. Undernutrition may be associated with prolonged length of stay and mechanical ventilation, infection, and mortality [10, 11]. Overnutrition may be also associated with prolonged mechanical ventilation and infection, as well as elevated glucose, urea, and hepatic function tests, which in turn are associated with increased morbidity [12].

Several predictive models have been proposed to prognosticate resting energy expenditure (REE). A retrospective validation of these models with actual measured energy expenditure [13] showed no correlation between predicted and measured energy expenditure, with a maximum agreement of 0.5 for some of the models [14, 15]. Moreover, the studies demonstrated that use of these models may result in nutritional recommendations which either over- or underestimate nutritional needs by 500 kcal or more, leading to over- or undernutrition, respectively.

Indirect calorimetry is the preferred method to more precisely guide caloric requirements for REE and is recommended by both ASPEN [16] and ESPEN guidelines [5]. However, because the availability of metabolic monitors for indirect calorimetry is limited, resting energy expenditure can be more readily derived from VCO2 (carbon dioxide production) obtained from the ventilator (REE = 8.2 × VCO2). While less accurate than indirect calorimetry measurement, this method of measuring REE is more precise than the use of predictive models [17]. In the absence of alternate methods, predictive models can be used with an awareness of their prognostic limitations.

**Fig. 1:**
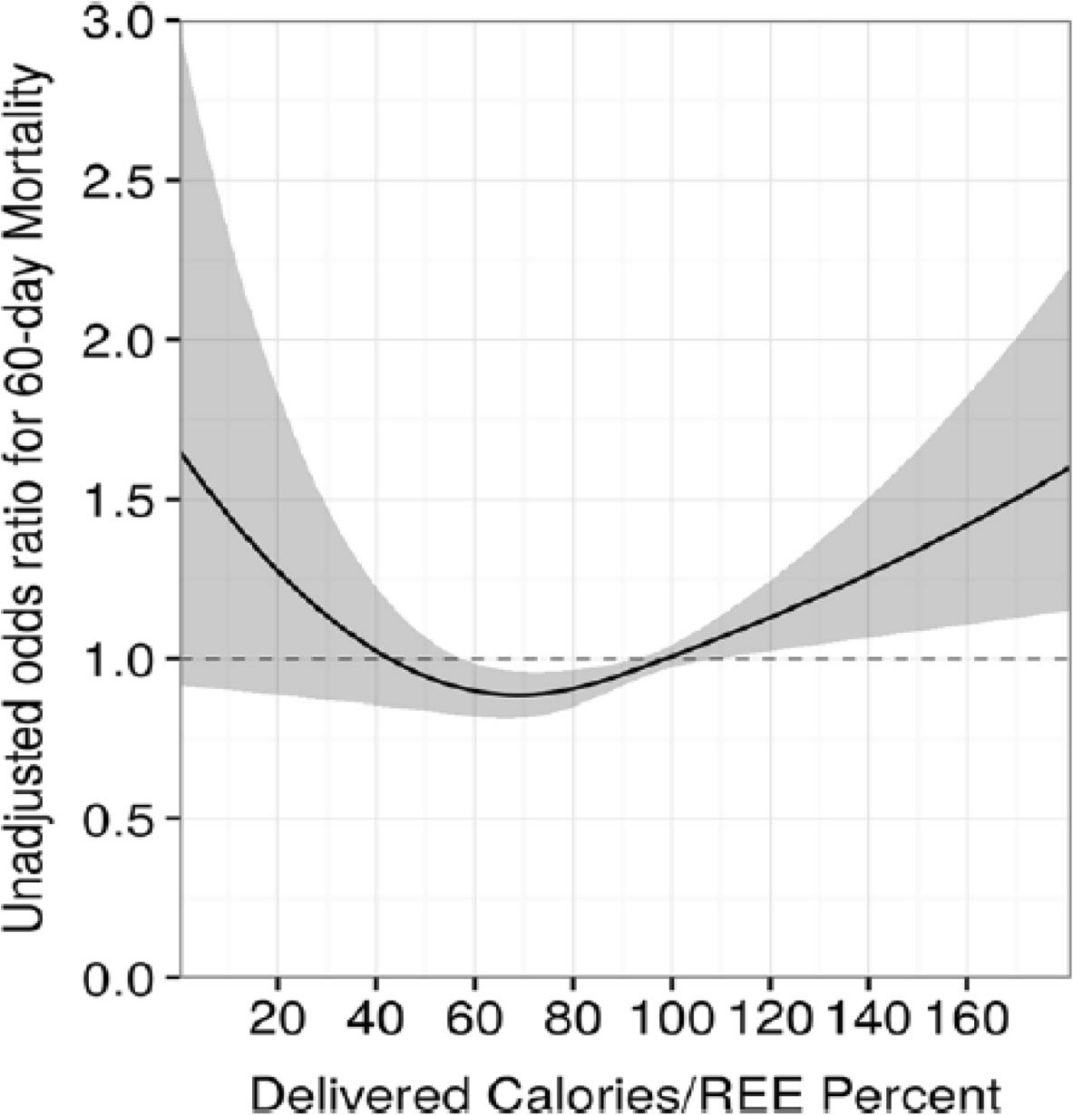
Survival according to calories delivered/energy expenditure ratio and according to protein administered. From [7] with permission

With respect to recommendations for optimum calorie intake, Zusman et al. [7] observed that providing between 70 and 100% of measured energy expenditure was associated with improved survival. It is remarkable to note that patients who received in excess of 100% of measured energy requirements showed an increase in mortality (Fig. 1), underscoring the importance of measuring REE and thereby avoiding overfeeding. While the study was retrospective and observational, it is the largest study to date comparing calorie intake to measured energy expenditure and outcome. However, prospective randomized trials are needed to confirm these findings.

Current guidelines recommend commencing enteral feeding early in the course of ICU stay, as this has been associated with a decrease in infections. Caloric energy targets should be reached within 3 days, provided that caution is taken to avoid overfeeding and the resultant increase in risk of complications [18]. If caloric goals cannot be reached through enteral feeding, parenteral nutrition can be administered between days 3 and 7 of ICU stay to supplement the lack of sufficient calories [5]. A balanced administration of macronutrients, including lipids together with carbohydrates, is recommended [5].

Special attention should be given to electrolyte status. Any significant decrease in potassium, phosphorus, or magnesium may endanger the patient and should be corrected promptly, coupled with a decrease in energy intake by 50% for the subsequent few days [19, 20]. Close monitoring of electrolyte status should continue throughout the ICU stay, as clinically significant disturbances can occur, not only at admission in severe malnourished patients, but also during the course of the ICU stay in well-nourished patients.

Recommendations for protein intake are 1.3 g/kg/day, combined with an exercise program [5]. Observational studies have shown that increased protein intake was associated with improved survival [21–23]. However, this mortality benefit was not confirmed in prospective randomized controlled trials [24], which demonstrated only improvements in renal function [25] or in muscle mass [26]. The improvement in renal function is related to an increase in renal glomerular reserve and varies with protein load and patient age [5]. Other studies have failed to demonstrate any clinical findings [27] favoring increased protein administration.

Protein intake recommendations are dependent upon patient clinical status. Sarcopenic patients with a substantial decline in muscle mass have increased mortality risk, and high protein diet may improve their survival [28]. However, in septic patients, protein intake does not appear to influence outcome [29], as evidenced in the EAT-ICU study [24] which did not show an improvement in septic patients receiving a high (1.4 g/kg/d) protein intake. A post hoc analysis, however, showed significantly improved survival in patients with normal renal function and high protein intake [30].

Exercise, such as with a cycloergometer, has been demonstrated to enhance short-term recovery, as evidenced by improved by 6 minute walk distance, force (?), and quality of life [31]. When mobilization is actively planned and implemented, a decrease in the duration of mechanical ventilation and length of stay has been observed [32]. However, outcomes are likely dependent upon duration and frequency as well as form of exercise. Moreover, the feasibility or appropriateness of aerobic exercise at bedside in ICU patients may limit its application.

Interestingly, early physical exercise during the first week of hospitalization for patients with septic shock was recently shown to preserve muscle fiber cross-sectional area [33]. Muscle fiber cross-sectional area (μm2) was preserved by exercise (− 25.8% ± 21.6% in control vs 12.4% ± 22.5% in the intervention group; *p* = 0.005) but not without. Markers of the catabolic ubiquitin-proteasome pathway were reduced at day 7 only in the intervention group. The excessive activation of autophagy observed after septic shock was suppressed without modification of the markers of anabolism and inflammation related to septic shock. These results imply an association between exercise and muscle preservation in septic patients and will require confirmation in larger studies.

### Nutrition in recovery from critical illness

During the recovery phase following liberation from mechanical ventilation, post-extubation dysphagia may occur [34] from 3% up to 60% of patients. Due to prolonged ventilation, many of these patients suffer from severe dysphagia, leading to decreased energy, micronutrient, and protein intake, as well as increased rates of pneumonia, reintubation, and mortality [35, 36]. Oral intake can be further impaired by post-extubation ventilatory support with high flow nasal canulla or other forms of non-invasive ventilation [37]. Paradoxically, a patient may achieve greater caloric intake through enteral feeding during mechanical ventilation than per os after extubation. In these circumstances, ESPEN guidelines recommend that nutritional needs be supplemented via nasogastric tube or parenteral feeding, to ensure adequate energy and protein supply [5]. This strategy should be combined with active deglutition rehabilitation.

Upon discharge from the ICU, the patient should be followed for the duration of the hospital stay by a nutrition team as length of stay may be prolonged and patients may suffer from additional loss of muscle and energy in the absence of good nutritional guidance and physical activity. A comprehensive multi-disciplinary approach to the critically ill patient from admission to the ICU through the hospital stay until discharge to rehabilitation should be implemented since increasing frailty while hospitalized can substantially impair the ability to achieve successful rehabilitation on discharge.

## Conclusions

Critically ill patients in the ICU are at significant risk for malnutrition. Feeding protocols not guided by an assessment of resting energy expenditure may result in under- or overfeeding. Indirect calorimetry is the preferred method to estimate an REE target to guide caloric intake. Protein intake should be planned to reach 1.3 g/kg/day. Emphasis should be placed on early enteral nutrition, and where not achievable, parenteral nutrition should be implemented with careful monitoring parameters to avoid overfeeding. Exercise can be an important adjuvant therapy to calorie and protein supplementation. As the patient progresses to recovery, efforts to maintain adequate nutritional intake should be continued to prevent under-nutrition secondary to dysphagia and poor oral intake. Nutritional support and supplementation tailored to the etiology and recovery stage of ICU patients can improve metabolic condition, decrease morbidity, and optimize long-term rehabilitation success.

## Abbreviations

ASPEN: American Society of Parenteral and Enteral Nutrition
ESPEN: European Society for Clinical Nutrition and Metabolism
ICU: Intensive care unit
PICS: Persistent Inflammatory Catabolism Syndrome
VCO2: Carbon dioxide production

## References

[1] Moore FA, Phillips SM, McClain CJ, Patel JJ, Martindale RG (2017). Nutrition support for persistent inflammation, immunosuppression, and catabolism syndrome. Nutr Clin Pract.

[2] Gardner AK, Ghita GL, Wang Z, Ozrazgat-Baslanti T, Raymond SL, Mankowski RT, et al. The development of chronic critical illness determines physical function, quality of life, and long-term survival among early survivors of sepsis in surgical ICUs. Crit Care Med. 2019; Epud ahead of publication. 10.1097/CCM.0000000000003655 PMC6422682 30664526

[3] Muscedere J, Waters B, Varambally A, Bagshaw SM, Boyd G, Maslove D (2017). The impact of frailty on intensive care unit outcomes: a systematic review and meta-analysis. Intensive Care Med.

[4] Kaukonen KM, Bailey M, Suzuki S, Pilcher D, Bellomo R (2014). Mortality related to severe sepsis and septic shock among critically ill patients in Australia and New Zealand, 2000-2012. JAMA.

[5] Singer P, Reitham Blaser A, Berger MM (2018). ESPEN Guidelines: nutrition in the ICU. Clin Nutr.

[6] Cuthbertson D (1970). Intensive-care-metabolic response to injury. Br J Surg.

[7] Zusman O, Theilla M, Cohen J, Kagan I, Bendavid I, Singer P (2016). Resting energy expenditure, calorie and protein consumption in critically ill patients: a retrospective cohort study. Crit Care.

[8] Puthucheary ZA, Rawal J, McPhail M, McPhail M, Connolly B, Ratnayake G (2013). Acute skeletal muscle wasting in critical illness. JAMA.

[9] Bendavid I, Singer P, Theilla M (2017). NutritionDay ICU: a 7 year worldwide prevalence study of nutrition practice in intensive care. Clin Nutr.

[10] Dvir D, Cohen J, Singer P (2005). Computerized energy balance and complications in critically ill patients: an observational study. Clin Nutr.

[11] Villet S, Chiolero RL, Bollmann MD, Revelly JP, Cayeux RNMC, Delarue J (2005). Negative impact of hypocaloric feeding and energy balance on clinical outcome in ICU patients. Clin Nutr.

[12] Braunschweig C, Sheean PM, Peterson SJ, Gomez Perez S, Freels S, Gurka D (2015). Intensive nutrition in acute lung injury: a clinical trial (INTACT). JPEN J Parenter Enteral Nutr.

[13] Zusman O, Kagan I, Bendavid I, Theilla M, Cohen J, Singer P. Predictive equations predictive equations versus measured energy expenditure by indirect calorimetry: a retrospective validation. Clin Nutr; 2018. Epub ahead of publication. 10.1016/j.clnu.2018.04.020 29776694

[14] Frankenfield DC, Coleman A, Alam S, Cooney RN (2009). Analysis of estimation methods for resting metabolic rate in critically ill adults. JPEN J Parenter Enteral Nutr.

[15] Tatucu-Babet OA, Ridley EJ, Tierney AC (2015). The prevalence of underprescription or overprescription of energy needs in critically ill mechanically ventilated adults as determined by indirect calorimetry: a systematic literature review. JPEN J Parenteral Enteral Nutr.

[16] Taylor BE, McClave SA, Martindale RG, Warren MM, Johnson DR, Braunschweig C (2016). Guidelines for the provision and assessment of nutrition support therapy in the adult critically ill patient: Society of Critical Care Medicine (SCCM) and American Society for Parenteral and Enteral Nutrition (A.S.P.E.N.). Crit Care Med.

[17] Stapel SN, de Grooth HJ, Alimohamad H, Elbers PW, Girbes AR, Weijs PJ, Oudemans-van Straaten HM (2015). Ventilator-derived carbon dioxide production to assess energy expenditure in critically ill patients: proof of concept. Crit Care.

[18] Reintam Blaser A, Starkopf J, Alhazzani W, Berger MM, Casaer MP, Deane AM (2017). Early enteral nutrition in critically ill patients: ESCIM clinical practice guidelines. Intensive Care Med.

[19] Doig GS, Simpson F, Heighes PT, Bellomo R, Chesher D, Caterson ID (2015). Restricted versus continued standard caloric intake during the management of refeeding syndrome in critically ill adults: a randomised, parallel-group, multicentre, single-blind controlled trial. Lancet Respir Med.

[20] Olthof LA, Koekkoek WACK, van Setten C, Kars JCN, van Blokland D, van Zanten ARH. Impact of caloric intake in critically ill patients with, and without, refeeding syndrome: a retrospective study. Clin Nutr. 2017; in press. 10.1016/j.clnu.2017.08.001 28866139

[21] Weijs PJ, Stapel SN, de Groot SD (2012). Optimal protein and energy mortality in mechanically ventilated critically ill patients: a prospective observational cohort study. JPEN J Parenter Enteral Nutr.

[22] Allingstrup MJ, Esmailzadeh N, Wilkens Knudsen A, Espersen K, Hartvig Jensen T, Wis J (2012). Provision of protein and energy in relation to measured requirements in intensive care patients. Clin Nutr.

[23] Nicolo M, Heyland DK, Chittams J, Sammarco T, Compher C (2016). Clinical outcomes related to protein delivery in a critically ill population: a multicenter, multinational observation study. JPEN J Parenter Enteral Nutr.

[24] Allingstrup MJ, Kondrup J, Wijs J, Claudius C, Pedersen UG, Hein-Rasmussen R (2017). Early goal-directed nutrition versus standard of care in adult intensive care patients: the single centre, randomised, outcome assessor-blinded EAT-ICU trial. Intensive Care Med.

[25] Doig GS, Simpson F, Bellomo R, Heighes PT, Sweetman EA, Chesher D (2015). Intravenous amino acid therapy for kidney function in critically ill patients: a randomized controlled trial. Intensive Care Med.

[26] Ferrie S, Allman-Farinelli M, Daley M, Smith K (2016). Protein requirements in the critically ill: a randomized controlled trial using parenteral nutrition. JPEN J Parenter Enteral Nutr.

[27] Heyland DK, Stapleton R, Compher C (2018). Should we prescribe more protein to critically ill patients?. Nutrients.

[28] Looijaard WG, Dekker IM, Stapel SN, Girbes AR, Twisk JW, Oudemans-van Straaten HM, Weijs PJ (2016). Skeletal muscle quality as assessed by CT-derived skeletal muscle density is associated with 6-month mortality in mechanically ventilated critically ill patients. Crit Care.

[29] Weijs P, Looijaard W, Beishuizen A, Girbes AR, Oudemans-van Staaten HM (2014). Early high protein intake is associated with low mortality and energy overfeeding with high mortality in non-septic mechanically ventilated critically ill patients. Crit Care.

[30] Zhu R, Allingstrup MJ, Perner A, Doig GS (2018). Nephro-Protective Trial Investigators Group: the effect of IV amino acid supplementation on mortality in ICU patients may be dependent on kidney function: post hoc subgroup analyses of a multicenter randomized trial. Post hoc Crit Care Med.

[31] Doiron KA, Hoffmann TC, Beller EM (2018). Early intervention (mobilization or active exercise) for critically ill adults in the intensive care unit. Cochrane Database Syst Rev.

[32] Schaller SJ, Anstey M, Blobner M, Edrich T, Grabitz SD, Gradwohl-Matis I (2016). Early, goal-directed mobilisation in the surgical intensive care unit: a randomised controlled trial. International Early SOMS-guided Mobilization Research Initiative. Lancet.

[33] Hickmaan CE, Castanares-Zapatero D, Deldicque L, Van den Bergh P, Caty G, Robert A, et al: Impact of very early physical therapy during septic shock on skeletal muscle: a randomized controlled trial. Intensive Care Med. 2018;46:1436–1443. 10.1097/CCM.0000000000003263 PMC6110624 29957714

[34] Macht M, White D, Moss M (2014). Swallowing dysfunction after critical illness. Chest.

[35] Kruser JM, Prescott HC (2017). Dysphagia after acute respiratory distress syndrome: another lasting legacy of critical illness. Ann Am Thorac Soc.

[36] Peterson SJ, Tsai AA, Scala CM, Sowa DC, Sheean PM, Braunschweig CL (2010). Adequacy of oral intake in critically ill patients 1 week after extubation. J Am Diet Assoc.

[37] Leder SB, Siner JM, Bizzarro MJ, McGinley BM, Lefton-Greif MA (2016). Oral alimentation in neonatal and adult populations requiring high-flow oxygen via nasal cannula. Dysphagia.

